# Electrospun Bioactive Wound Dressing Containing Colloidal Dispersions of Birch Bark Dry Extract

**DOI:** 10.3390/pharmaceutics12080770

**Published:** 2020-08-14

**Authors:** Francis Kamau Mwiiri, Johanna M. Brandner, Rolf Daniels

**Affiliations:** 1 Department of Pharmaceutical Technology, Eberhard Karls University, Auf der Morgenstelle 8, 72076 Tuebingen, Germany; f.kamau@gmx.de; 2 Department of Dermatology and Venerology, University Hospital Hamburg-Eppendorf, Martinistraße 52, 20246 Hamburg, Germany; brandner@uke.de

**Keywords:** electrospinning, PVA, birch bark extract, wound dressing, phospholipids

## Abstract

Novel birch bark dry extract (TE)-loaded polyvinyl alcohol (PVA) fiber mats intended for wound therapy were developed through an electrospinning process. Colloidal dispersions containing TE as the active substance were prepared by the high-pressure homogenization (HPH) technique using hydrogenated phospholipids as stabilizer. Subsequently, the colloidal dispersions were blended with aqueous PVA solutions in the ratio of 60:40 (wt.%) and electrospun to form the nanofiber mats. Fiber morphology examined using scanning electron microscopy (SEM) indicated that fibers were uniform and achieved diameters in the size range of 300–1586 nm. Confocal Raman spectral imaging gave good evidence that triterpenes were encapsulated within the electrospun mats. In vitro drug release and ex vivo permeation studies indicated that the electrospun nanofibers showed a sustained release of betulin, the main component of birch bark dry extract, making the examined dressings highly applicable for several wound care applications. Ex vivo wound healing studies proved that electrospun fiber mats containing TE accelerated wound healing significantly more than TE oleogel, which was comparable to an authorized product that consists of TE and sunflower oil and has proved to enhance wound healing. Therefore, our results conclude that the developed TE-PVA-based dressings show promising potential for wound therapy, an area where effective remedy is needed.

## 1. Introduction

The human skin serves as a barrier between the body and the environment. Therefore, it is prone to microbial, thermal, mechanical, and chemical threats, which can cause acute or chronic wounds. Triterpenes have been shown to improve wound healing recovery by inducing cell migration, cell proliferation, and collagen deposition [1]. Outer birch bark extracts have a high content of triterpenes, which are known for various pharmacological properties such as anti-inflammatory, antimicrobial, antiviral, anticancer activity, and wound healing effects [2,3,4,5,6,7,8,9,10]. A well characterized commercially used triterpene dry extract from the outer bark of birch (TE) contains about 80% (*w/w*) betulin as the main component and is obtained by accelerated solvent extraction with n-heptane [11]. Other disclosed triterpenes of the dry extract include lupeol (LU), erythrodiol (ER), betulinic acid (BA), and oleanolic acid (OA) as shown in Table 1 [12]. However, the low solubility of these triterpenes in polar and nonpolar solvents may lead to a poor bioavailability, which might limit their therapeutic application [8,13].

A study conducted by Ebeling et al. showed the molecular mechanism of the effects of birch bark extract on wound healing properties in human primary keratinocytes and porcine ex vivo wound healing models. They showed that TE and betulin mainly accelerated reepithelialization in a porcine ex vivo wound healing model after wound treatment with TE-oleogel (10% TE, 90% sunflower oil). Beyond that, they found out that TE led to upregulation of proinflammatory mediators such as COX-2 and IL-6, which play a key role in wound healing und epidermal barrier repair [6,14]. Additionally, the effects of TE-based formulations have been already investigated in vivo on different types of wounds including dystrophic epidermolysis bullosa where treatments promote wound healing and was found out to be safe and well tolerated [15,16,17]. It is good to note that TE together with sunflower oil (SO) supports wound healing better than in combination with other oils as examined by Steinbrenner et al. [8]. At the moment, there are a few available topical formulations containing these triterpenes, including water-in-oil foams [9], cosmetic water-in-oil creams, and an oleogel consisting of TE and sunflower oil that received European marketing authorization in January 2016 [18].

The electrospinning technique is a simple and versatile process for preparing fibers having a diameter from few micrometers down to several nanometers [19,20]. The resulting nanofibers have special features, such as high surface area to volume ratio, and can form mats/fleeces with high porosity which makes them attractive materials for wound dressing [21,22]. Beyond that, electrospun fibrous scaffolds mimic the structure of the native extracellular matrix (ECM) and hence facilitate cell proliferation, improve gaseous exchange, removal of exudate, and act as a physical barrier against entry of microorganisms during wound healing and regeneration of damaged tissues [21,23,24,25,26].

Polymeric nanofibers made from biodegradable and biocompatible synthetic or natural polymers have been utilized to develop drug delivery systems to treat various ailments. One of the potential areas to use them is medicated wound dressing [21,23]. PVA is a biocompatible and biodegradable hydrophilic polymer with good chemical and mechanical properties, and has been approved by the U. S. Food and Drug Administration (FDA) for different biomedical and pharmaceutical applications [27,28]. For instance, PVA has been used to create hydrogels for wound dressing [29] or electrospun together with active substances, such as silver nanoparticles [30], and curcumin [31], to produce wound dressings. Several wound dressings in the market containing active agents such as hydrocolloids or silver dressings often used on wound healing have shown negative effects such as allergic contact dermatitis [32], and use of dressings loaded with antibiotics has risk of developing antimicrobial resistance [33]. Our interest in this study is to develop a dressing loaded with birch bark extract with a sustained drug release that is suitable for human use on wound healing with effective therapy even after long-term usage. To satisfy this requirement, we ensured that all the components used in this study are biocompatible and biodegradable when applied to the human body, such that an invasive and/or traumatic intervention for dressing removal, like in the case of deep wounds, is unnecessary. Furthermore, sustained drug release could lead to less frequent dressing changes, which are painful, especially in treatment of chronic wounds, and thereby resulting in better patient compliance. In addition to that, our concept allows one to avoid organic solvents and uses water as the only solvent throughout the whole processing of the wound dressing. This guarantees not only that the solvent is nontoxic and affordable, but also that it is eco-friendly and can thus be regarded as green electrospinning. However, encapsulation of birch bark dry extract into nanofibers may present a challenge. For instance, earlier studies showed that due to their unique structure, the particle sizes of the dry extract cannot be grinded by common techniques to reach sufficiently small particles even when high energy dispersion techniques, e.g., sonication or high-pressure homogenization, were used with aqueous and organic suspensions of the material [34]. This was solved through processing of oil in water (O/W) colloidal dispersions with birch bark dry extract as the active substance, phospholipids as stabilizer, sunflower oil, and the water phase through a step-wise homogenization process. Hydrogenated soybean phosphatidylcholine (HSPC) types of phospholipids containing mainly esterified stearic and palmitic acid enhanced the producibility of colloidal dispersions with particle sizes < 1 µm and enabled miscibility and stabilization of the lipophilic components in the hydrophilic PVA matrix prior to electrospinning.

The purpose of this study was to (1) develop wound dressing materials with incorporated colloidal dispersions of TE and to (2) study their efficiency related to wound healing performance and (3) drug release. The surface properties of electrospun fiber mats were characterized by using SEM, thermal analysis with differential scanning calorimetry (DSC) and confocal Raman spectral imaging for visualization of all components within the fiber mats. We also conducted ex vivo permeation and in vitro release studies using Franz diffusion cell. The efficacy of the developed wound dressings was evaluated through an ex vivo wound healing assay. Here, low and highly TE-loaded dressings were tested and compared to an oleogel similar to the authorized product. To the best of our knowledge, there are no reports focusing on the development of this kind of wound dressing; therefore, this will be the first polymer-based wound dressing containing birch bark dry extract aimed for future potential use in wound therapy.

## 2. Materials and Methods

### 2.1. Materials

PVA with a molecular weight of 146–186 kDa was purchased from Sigma Aldrich (Steinheim, Germany), hydrogenated phospholipids from soybean lecithin (Phospholipon 90 H) were supplied by Lipoid GmbH (Ludwigshafen, Germany). SO was purchased from Caesar & Loretz GmbH (Hilden, Germany). Birch bark extract was obtained from Amryt AG (Niefern-Öschelbronn, Germany). Reverse osmosis water (ELGA Labwater, Celle, Germany) was used for the preparation of all solutions. Parafilm^®^ was from Bemis Company Inc., (Oshkosh, WI, USA). Whatman Nuclepore polycarbonate membrane filters were purchased from Sigma-Aldrich (Steinheim, Germany). Pig ears for permeation studies were obtained from the Department of Experimental Medicine at the University of Tuebingen [35] and for wound healing studies from a slaughter house in Schleswig-Holstein after slaughtering the pigs for human consumption.

### 2.2. Preparing the Colloidal Dispersions

Three different dispersions were prepared with varying concentrations of TE as shown in Table 2. Dispersions 1 and 2 (D1 and D2) were necessary to investigate the impact of high or low TE concentration on wound healing, whereas dispersion 3 (D3) was used as a placebo. It is important to mention that it was only practically possible to produce dispersions consisting of high TE (5%) loading by use of high concentrations of PL90H (8%). That is why 8% PL90H was used in preparation of dispersion consisting 5% TE. Dispersion containing 2.5% PL90H was the optimized colloidal dispersion from our previous study. Briefly, during preparation of each formulation, a predispersion was prepared by first dispersing Phospholipon 90H (PL90H) and TE in water at 70 °C for 30 min under magnetic stirring at 400 rpm using Heidolph MR 3001 K magnetic hotplate stirrer (Schwabach, Germany). Then, this mixture was homogenized for 5 min using a rotor-stator system (Ultra Turrax T25, IKA, Staufen, Germany), at 9500 rpm. Thereafter, the formed dispersion was added into the SOand homogenized for 3 min. Finally, the predispersion was homogenized using a high-pressure homogenizer (Emulsiflex C-3, Avestin, Mannheim, Germany) for eight cycles at a pressure of 100 MPa and their particle sizes measured with a Zetasizer Nano-ZS (Malvern Instruments, Herrenberg, Germany).

### 2.3. Preparation of Electrospinning Solutions

The 12 wt.% PVA solution was prepared by dissolving PVA in water at 90 °C under magnetic stirring for 5 h. The solution was allowed to cool to room temperature and used the following day. Subsequently, for colloidal dispersion-PVA electrospinning experiments, the above aqueous dispersions (D1–D3) were blended with a PVA polymer solution in the ratio of 60:40 using a magnetic stirrer for 2 h at 40 °C to form a homogeneous solution. Samples were then cooled down to room temperature prior to electrospinning.

### 2.4. Electrospinning of Nanofibers

The electrospinning setup purchased from Nanolab Instruments Sdn. Bhd., Subang Jaya, Malaysia consisted of a syringe pump holding a 5 mL plastic syringe with a blunt-end needle (18-gauge), a grounded rotating collector, and a high-voltage power supply. Electrospun fibers were prepared from solutions and dispersions as listed in Table 3. Pure polymer solution serves as a reference mainly for the physicochemical characterization. F1 represents the placebo formulation. F2 and F3 were chosen to investigate the effect of high and low TE concentration. The syringe was filled with the solution and electrospun at a target collector distance of 10 cm and an applied voltage of 15 kV. Solutions were pumped through the syringe at a flow rate of 0.5 mL/min and the rotating speed of the collector was fixed at 1000 rpm. All electrospinning studies were carried out at ambient temperature (24 °C) and a relative humidity of 45%.

### 2.5. Characterization of Nanofiber Morphology

Fiber morphology was analyzed through SEM, Zeiss DSM 940 A, (Carl Zeiss GmbH, Oberkochen, Germany). Fiber mats of 0.5 cm × 0.5 cm sizes were placed of a conductive double-sided tape and sputter coated with gold using Biorad E 5100 Sputter Coater (Bio-Rad GmbH, Munich, Germany) at 2.1 kV and 20 mA for 240 s and imaged using the microscope. Thereafter, the average fiber diameters were determined from the SEM images by randomly selecting a minimum of 30 segments and their diameters calculated using ImageJ software (National Institute of Health, Bethesda, MD USA). For fiber diameter distribution, numbers of fiber diameters were converted into their percentage total values and plotted against grouped fiber diameter. The thicknesses of the fiber mats were measured with a digital micrometer using both magnetic induction and eddy current methods (Dualscope FMP20, Helmut Fischer GmbH, Sindelfingen Germany) taking the average of 15 measurements at randomly selected places.

### 2.6. Confocal Raman Spectral Imaging

Raman images of wound dressings were acquired using an alpha 500R Raman microscope (WiTec GmbH, Ulm, Germany) equipped with a DV401-BV charge coupled device (CCD) detector, connected via an optical fiber to a UHTS 300 spectrometer and a 532 nm laser excitation source. A 100× air objective (EC Epiplan-Neofluor, Carl Zeiss, Oberkochen, Germany) with a numerical aperture of 0.9 was used to view the sample while the laser intensity was adjusted to 35 mW. The spectral range covered the fingerprint region between 710 and 1820 cm^−1^ and region between 2700 and 3580 cm^−1^. Spectra of all single components (PL90H, SO, PVA and TE) were first collected and used to monitor the presence of each component in the wound dressing. Subsequently, nanofiber mats were presented to the microscope on glass slides (VWR-International, Darmstadt, Germany) without the use of a coverslip. Image scans of wound dressings using 25 µm × 25 µm area of the surface were taken at an integration time of 0.01 s. Henceforth, color-coded images were obtained by initial cosmic ray removal and spectral background subtraction using the WiTec Project data analysis software 4.1 (WITec GmbH, Ulm, Germany, 2016). By assigning the spectrum of each component to a color, the distribution of all components within the formulation can be indicated, resulting in a color-coded image of the scanned area [36].

### 2.7. Differential Scanning Calorimetry (DSC)

Differential scanning calorimetry (DSC) studies of the produced nanofiber mats were carried out using a DSC 820 differential scanning calorimeter (Mettler-Toledo GmbH, Gießen, Germany). The DSC was calibrated for temperature and enthalpy using indium as a standard. Samples of 4–7 mg were sealed in an aluminium pan with one pinhole in the lid. The furnace was purged with nitrogen at a flow rate of 80 mL/min. The DSC scans were obtained by heating in the range of 25,250 °C at a rate of 10 °C/min.

### 2.8. Skin Permeation and In Vitro Drug Release Studies

Both permeation and release studies were performed using modified vertical Franz diffusion cells (Gauer Glas, Püttlingen, Germany) with a receptor volume of 12 mL. For in vitro release studies, synthetic polycarbonate membranes with a pore size diameter of 0.4 µm were used to separate donor and receptor compartments. For permeation studies, pig ears were obtained from the Department of Experimental Medicine of the University Hospital Tuebingen. The live animals were kept at the Department of Experimental Medicine and sacrificed during their experiments, with the approval of the ethics committee of the University Hospital Tuebingen. The ears were delivered directly after the death of the animals. The Department of Pharmaceutical Technology is registered for the use of animal products at the District Office of Tuebingen (registration number: DE 08 416 1052 21). Fresh pig ears were first cleaned with isotonic saline using cotton balls and after postauricular skin excision, they were wrapped in aluminium foil and stored at −30 °C until use. During the day of experiment, the skin samples were thawed at room temperature, skin strips of 3 cm width were made and pinned to a block of Styrofoam precovered with aluminium foil. Thereafter, wounded skin samples were prepared according to [9] by a skin grafting method using a Dermatom (Dermatom GA 630, Aesculap AG & Co. KG, Tüttlingen, Germany). The skin was first “wounded” through removal of the outermost layers of the skin with a thickness of 0.2 mm using Dermatom. Subsequently, the remaining skin was then dermatomed to a thickness of 0.4 mm. From the prepared porcine skin, a specimen was punched to obtain discs of 25 mm in diameter using a circular hole punch (Eduard Gottfried Ferne, Remscheid, Germany) [37].

The synthetic membrane/dermal porcine skin was mounted between the compartments of diffusion cells, and the effective diffusion area was 1.77 cm^2^. For both in vitro release and ex vivo permeation experiments studies, samples of 20 mg fiber mats (F2 and F3), exactly weighed, were used. Consequently, all samples were loaded in the donor compartments and covered with parafilm to prevent solvent evaporation. A mixture of ethanol and water, 50:50 (*v/v*), was used as the receptor medium and the diffusion cells were maintained at 32 °C under constant magnetic stirring at 500 rpm. At predetermined time intervals, samples of 1 mL were taken from the receptor compartment and replaced with the same volume of fresh prewarmed receptor medium to maintain sink condition. The samples removed were analyzed directly using the HPLC-UV method as described below. The experiments were conducted in triplicate.

### 2.9. Entrapment Efficiency

The amount of betulin entrapped within fiber mats was estimated by dissolving approximately 20 mg of electrospun fiber mat, exactly weighed, in 30 mL ethanol:water (50:50 *v/v*) solution by means of sonication at 65 °C (Sonorex RK 31H, Bandelin, Berlin). Samples were then filtered using hydrophilic polyethersulfone (PES) syringe filters (Macherey-Nagel, Düren, Germany), and the total amount of drug extracted was quantified by High Performance Liquid Chromatography with Ultraviolet detection (HPLC-UV). The percentage entrapment efficiency (%EE) was calculated by the following Equation (1):

(1) $$\%\;\mathrm{EE}=\frac{\mathrm{Total}\;\mathrm{mass}\;\mathrm{of}\;\mathrm{drug}\;\mathrm{extracted}\;\mathrm{from}\;\mathrm{nanofiber}}{\mathrm{Mass}\;\mathrm{of}\;\mathrm{total}\;\mathrm{drug}\;\mathrm{added}\;}\;\times \;100\%$$

### 2.10. Betulin Permeation/Release Kinetics Studies

The release/permeation kinetics were estimated by linear regression analysis of the in vitro release and ex vivo permeation data using various mathematical models [38]. The four models included zero order (Equation (2)), first order (Equation (3)), Korsmeyer–Peppas (Equation (4)), and Higuchi (Equation (5)) and the mathematical model that best fitted the kinetic release profile was selected based on the highest coefficient of determination, R^2^. In all equations, M_t_ represents the amount of betulin released at time t.

Zero-order model, where k_0_ is the zero-order release constant:

M_t_ = k_0_t (2)

First order model where K_1_ is the first order release rate constant:

In (1 − M_t_) = −K_1_t (3)

Korsmeyer–Peppas model:

Mt = kt^n^ (4)

where K is the Korsmeyer–Peppas constant, which is connected to the characteristics of the delivery system and the encapsulated drug. The n is the diffusional exponent that shows the drug release mechanism; where *n* equal to 0.45 represents a Fickian diffusion mechanism and 0.45 < *n* < 1 is referred to as a non-Fickian diffusion mechanism (anomalous transport) in which both Fickian diffusion and Case-II transport occurs [39].

Higuchi model, where k is Higuchi constant:

Mt = kt^0.5^ (5)

### 2.11. HPLC Analysis

Betulin quantification was performed using an LC-20A prominence HPLC system (Shimadzu, Kyoto, Germany). The mobile phase, a mixture of acetonitrile and water with the addition of 0.1% (*v/v*) phosphoric acid, was used as a gradient system for chromatographic separation. For efficient separation, gradient conditions were used according to the isocratic/gradient modes developed by Armbruster et al. [40]. A Nucleosil 100-5 C18 EC 125/4 column with a precolumn Universal RP EC 4/3 (Macherey-Nagel, Düren, Germany) was kept at 40 °C and a flow rate of 1.2 mL/min was used. A sample volume of 100 µL was injected and the peaks were detected at 210 nm. The retention time of betulin was approximately 7.5 min.

### 2.12. Ex Vivo Wound Healing Assay

In order to characterize the wound healing efficiency, a porcine ex-vivo wound healing assay was performed as described elsewhere [8]. Briefly, pig ears were immediately delivered after slaughtering for human consumption to the laboratory, cleaned, and disinfected. Thereafter, 6 mm punch biopsies were taken from the plicae of the ears and fat and subcutis were removed. Consequently, wounds were generated by the removal of the epidermis and upper dermis in a central area of 7.1 mm^2^. Then, the so formed ex vivo wound healing model was placed dermis-down on gauze in culture dishes and incubated at the air–liquid interface with Dulbecco’s modified Eagle’s medium supplemented with hydrocortisone, 2% fetal calf serum, penicillin, and streptomycin. The tested sample groups were divided into six groups (*n* = 10): (i) untreated control, (ii) TE-oleogel, (iii) pure PVA mat (iv) F1, (v) F2, and (vi) F3 fiber mats. Subsequently, sections of the electrospun fiber mats (4 mm in diameter)/5 µL of the oleogel were immediately applied after wounding, and the models were incubated for 48 h at 37 °C and 5% CO_2_. Further steps involved shock freezing, preparations of cryostat sections of the central parts of the wound healing models, and staining with hematoxylin and eosin. Wound healing progress (reepithelialization) was assessed by measuring the distance between the wound margin and the tip of the regenerated epidermis using a Leica DMLS microscope (10×), a Leica MC 170 HD CCD camera, and the Leica LAS V4.9 software (Leica, Wetzlar, Germany, 2017). Means of left and right wound margins were calculated.

### 2.13. Statistical Analysis

All the experiments unless mentioned otherwise were carried out in triplicate and the results were expressed as mean ± standard deviation (mean ± SD). Statistical analysis of the acquired data was performed by Student’s *t*-test and analysis of variance (ANOVA). *p*-value less than 0.05 was considered statistically significant, and where significance has been proven, it is indicated by * *p* < 0.05, ** *p* < 0.01, and *** *p* < 0.001.

## 3. Results and Discussion

### 3.1. Fiber Preparation and Morphological Characterization Using SEM

Preparation of the electrospun fibers building the wound dressing mats followed the optimized protocol as given in Section 2.4. In our previous preliminary studies, we varied all typical parameters that might have an impact on the fiber’s properties. Parameters investigated included (1) flow rate, which was varied between 0.1, 0.5, and 2 mL/h, (2) needle tip-to-collector distances were in range of 5, 10, and 20 cm, (3) speed of the collector was fixed at 1000 rpm, and (4) voltage was varied at 7, 15, and 20 kV. The electrospinning parameters used to manufacture fiber mats as highlighted in Section 2.4 were optimized to a flow rate of 0.5 mL/h, needle tip-to-collector distance of 10 cm, and an applied voltage of 15 kV.

**Figure 1:**
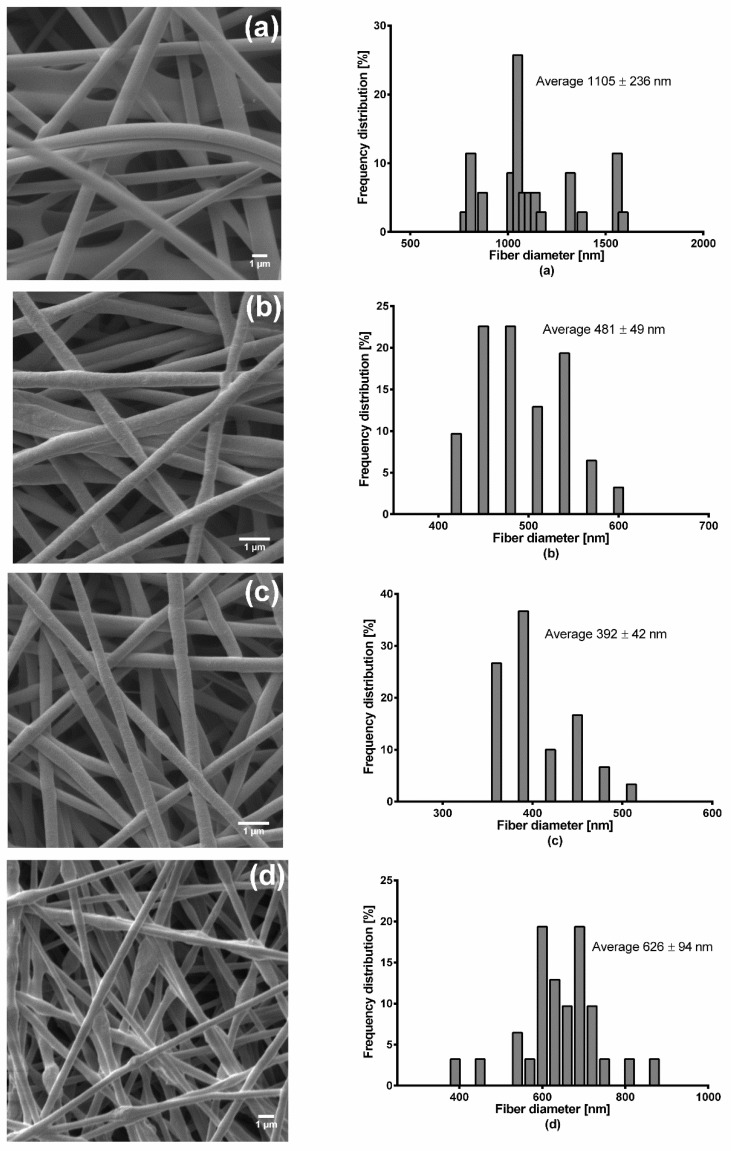
SEM micrographs and fiber diameter distribution of (**a**) pure PVA, (**b**) F1 (**c**) F2, and (**d**) F3 electrospun fiber mats.

Figure 1 shows SEM images of the electrospun wound dressings and the correspondingly measured fiber diameters. Pure PVA fiber mats had a thickness of about 51.67 ± 4.9 µm, F1 of 100 ± 9.09 µm, F2 of 39.59 ± 3.4 µm, and F3 exhibited the highest thickness of 110 ± 5.4 µm. Pure PVA electrospun fibers were randomly oriented with smooth surface and a few interconnected web-like structures in several points, while their fiber diameter ranged from approximately 750 to 1600 nm. The fiber diameter of F1 was between 390 and 600 nm without formation of beads. F2 fibers were observed as smooth and uniform with fiber diameters of 340 to 500 nm without beads. On the other hand, F3 fibers were observed as uniform but rough surfaces with presence of interconnected beads and their fiber diameter ranged from 350 to 900 nm.

**Figure 2:**
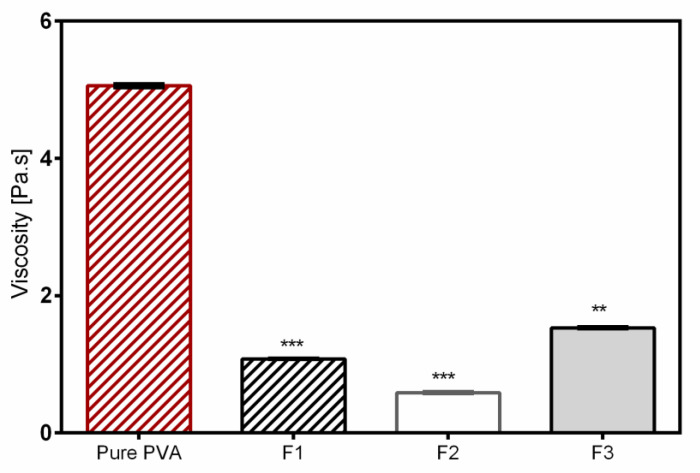
Viscosity of nonloaded and TE-loaded PVA solutions. * statistically significant, ** *p* < 0.01; *** *p* < 0.001 vs. pure PVA.

The results clearly show that not only the morphology of electrospun fibers, but also the resulting fiber diameter was changed after PVA was blended with the aqueous dispersions. In general, the addition of colloidal dispersions resulted in a decrease of the average fiber diameter due to the reduced viscosity of polymeric solutions shown in Figure 2. However, the increase of TE concentration in the mixture influenced the structure of the fibers, and the average diameter of the nanofibers also increased (Table 4). The observed web-like interconnected structures/beads were due to high viscosity, which caused resistance to jet stretching during the electrospinning process. In addition, it was very difficult to process F3 with a high amount of TE (2%) because unstable Taylor cone and discontinuous jet during electrospinning with intermediate clogging of the needle occurred. The discontinuous jet formation led to loss of the electrospinning solution, meaning that much drug was also lost in the process. Therefore, lower amounts of constituents in the colloidal dispersions led to reduction of viscosity, which resulted in better and stable stretching of the jet with an overall good performance during the electrospinning process even over a long production run (4 h). As a result, improved fiber morphology with smooth, thinner fibers was observed [41,42]. The two formulations showed good drug entrapment efficiencies of about 76% for F2 and 69% for F3.

### 3.2. Differential Scanning Calorimetry Analysis

**Figure 3:**
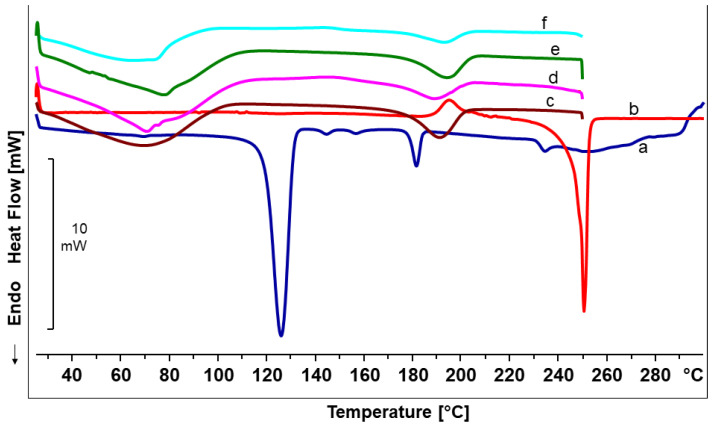
DSC thermograms for (a) Phospholipon 90H, (b) birch bark extract (TE), (c) Pure PVA mat, (d) F1, (e) F2, and (f) F3 fiber mats.

DSC measurements of the pure constituents of the electrospun fiber mats as well as of the final fiber mats were performed to observe the effect of the addition of colloidal dispersions in PVA matrix; their thermograms are presented in Figure 3. The DSC thermogram of the TE extract showed the melting process of a metastable modification at the beginning, accompanied by subsequent exothermic recrystallization in a more stable form, which melts at about 248 °C [43]. PL90H showed a major endothermic peak at around 119 °C where a partial melting of side chains as well as phosphatidylcholine transition to a thermotropic liquid crystalline state occurs. The peaks at around 141 °C, 153 °C, and 178 °C were thought to show further phase transitions, and lastly, the peak at 231 °C is where melting of phosphatidylcholine takes place [44]. The DSC thermograms of the electrospun products are characterized by two major endothermic peaks, one broader band between 70–100°C associated with evaporation of water, and a melting peak at 177 °C. This melting peak is in good accordance with the melting of pure PVA fibers (Table 5). Interestingly, the electrospun fibers show neither the melting peaks and phase transitions of the phospholipid, nor the melting peak of the TE. This indicates that the colloidal dispersions embedded in nanofibers were present mostly in the amorphous state. However, a final decision on the crystallinity requires further analytical methods, e.g., X-ray diffraction [45,46].

### 3.3. Confocal Raman Spectral Imaging

**Figure 4:**
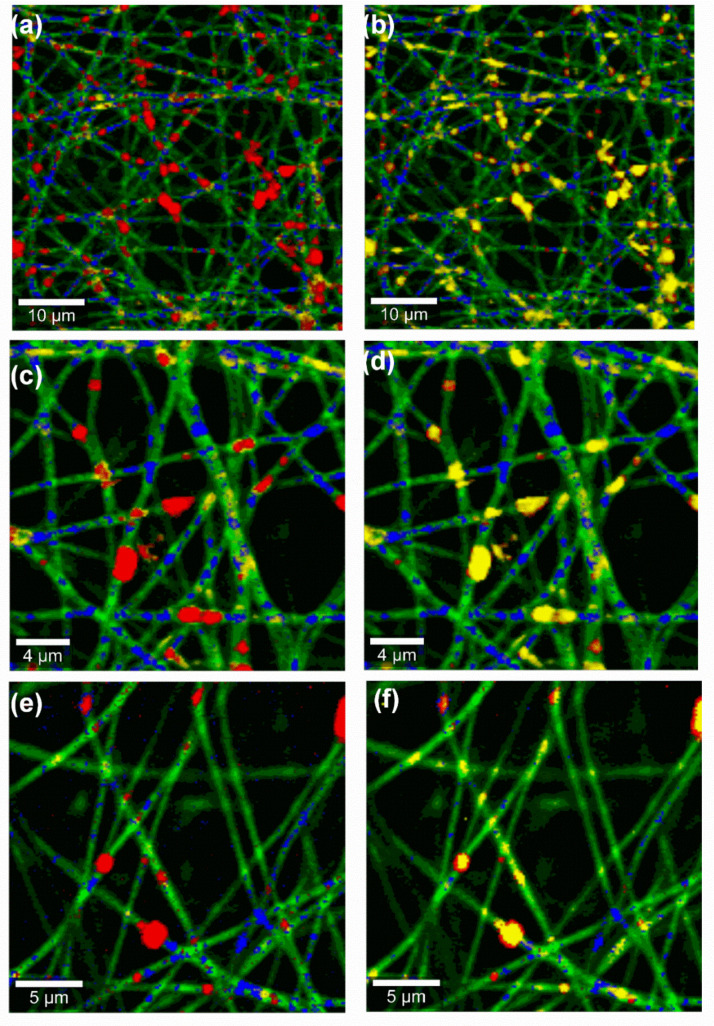
Confocal Raman microscopic color-coded images of TE-loaded fiber mats from (**a**–**d**) F3 (**e**), and (**f**) F2, Color code: Red: TE, Blue: PL90H, Green: PVA, Yellow: Sunflower oil; stack order of color code left column: green, yellow, blue, red; stack order of color code right column: green, red, blue, yellow.

The obtained Raman images of the nanofiber mats of F2 and F3 are shown in Figure 4. By observing the red spots, these color-coded images clearly show that F3 contained higher amount of the incorporated TE. TE (red spots) is found predominantly colocated with the oil phase (yellow spots), demonstrating that the extract has high affinity to sunflower oil. Although spatial resolution of Raman microscopy is in the submicron range and not on the molecular level, the images give, as expected, clear hints that the phospholipon 90H (blue spots) forms an interfacial layer between the lipophilic components (TE and sunflower oil) and PVA (green). Consequently, this will ensure better encapsulation as well as stabilization of the two lipophilic substances within the hydrophilic PVA matrix. Indeed, the color-coded images not only show homogeneous TE distribution throughout the fiber mats, but also strengthens a good encapsulation of TE within the nanofiber mats. Accordingly, confocal Raman microscopy allowed the investigation of the spatial distribution of all components within the fiber mats.

### 3.4. In Vitro Release Studies

**Figure 5:**
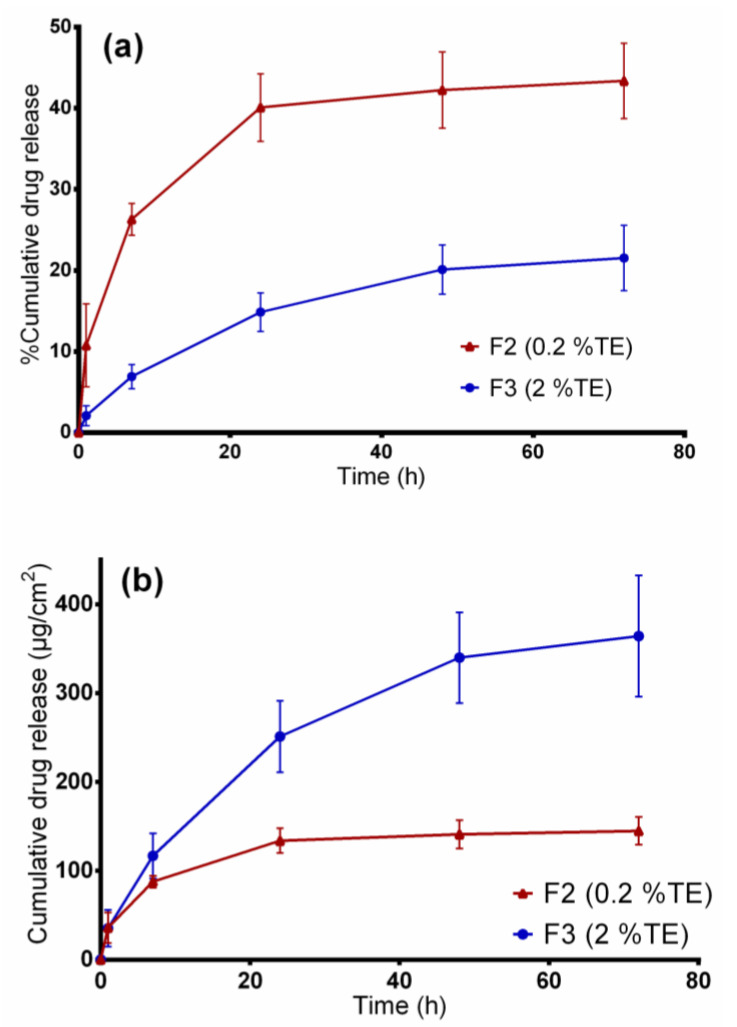
In vitro drug release studies. (**a**) Percentage of released betulin and (**b**) amount of betulin released (µg/cm^2^) from TE-loaded wound dressings.

Figure 5 shows the cumulative betulin release from PVA-based wound dressings for a period of 72 h at 32 °C represented in percent and µg/cm^2^ for the two TE-containing wound dressings. The release in µg/cm^2^ units could be vital for a possible clinical therapy, which makes it easier to calculate the dose needed based on the size of the wound dressing. Besides, the release in percentage would thereby be helpful to estimate the encapsulation efficiency. By observing the percentage amount released after 3 days, 44% of betulin was released from the F2 nanofibers whereas only 22% was released from F3 fiber mats. Although the amount of TE incorporated in F3 blend was high, this formulation shows a slower percentage drug release than F2 (*p* < 0.05) where a faster release is observed. The reason behind this was F3 blend contained components at very high concentrations (3.2% PL90H, 4% SO), which slowed the release of the drug, whereas the F2 blend had only 1% PL90H and 0.4% SO, hence quicker release of the drug from the hydrophilic matrix. Obviously, the amount released in µg/cm^2^ from F3 formulation was higher than F2 blend due to large differences in the amount initially incorporated. From these results, we can conclude that, initially, the drug is being released faster from the wound dressings, followed by a sustained release manner.

Table 6 shows the modelling results obtained from the four kinetic models. On comparing the R^2^ values of the two formulations, it was found that betulin release from the electrospun PVA wound dressings closely fitted Korsmeyer–Peppas model as this model showed higher R^2^ values followed by Higuchi square root model than other models. From the Korsmeyer–Peppas model, n values were found to be between 0.42 and 0.62 for both dressings, indicating that betulin release from PVA/TE fiber mats can be largely described as matrix diffusion [47,48].

In addition, the obtained results show that, by regulating the sunflower oil and PL90H concentrations in both formulations, betulin release can be controlled. Several mechanisms could be responsible for betulin’s controlled release. In our previous study, we observed through interfacial tension measurements that PL90H and TE strongly interact with each to form a stable film. Moreover, studies have shown that triterpenes interact with PL and fluidize lipid membranes controlled by their free polar groups where tetracyclic triterpenes (e.g., cortisol) tend to be located at the head group, whereas pentacyclic ones (e.g., erythrodiol), being more hydrophobic, are incorporated deeply in the lipid bilayers [49]. The ratios between PL90H:TE were 2:0.4 for F2 and 6.4:4 for F3 which demonstrates that increasing the concentration of PL90H reduces rate of drug release. On the other hand, the F2 contained lower concentrations of sunflower oil and PL90H which allows release medium to easily penetrate through the hydrophilic matrix, hence allowing faster betulin release. Another possible reason is that the F2 dressing had thinner fibers, with an average fiber diameter of 392 nm, than the thicker fibers of F3 (626 nm) where betulin has to travel a longer distance through the polymeric matrix, leading to a slower release [50]. It is also known that through emulsion electrospinning, core–shell nanofibers can be produced while designing controlled release drug delivery systems such that the drug release rate can be modulated, for example, by just adjusting the oil phase and the water phase of the emulsions [51].

### 3.5. Ex vivo Permeation Through Wounded Skin

**Figure 6:**
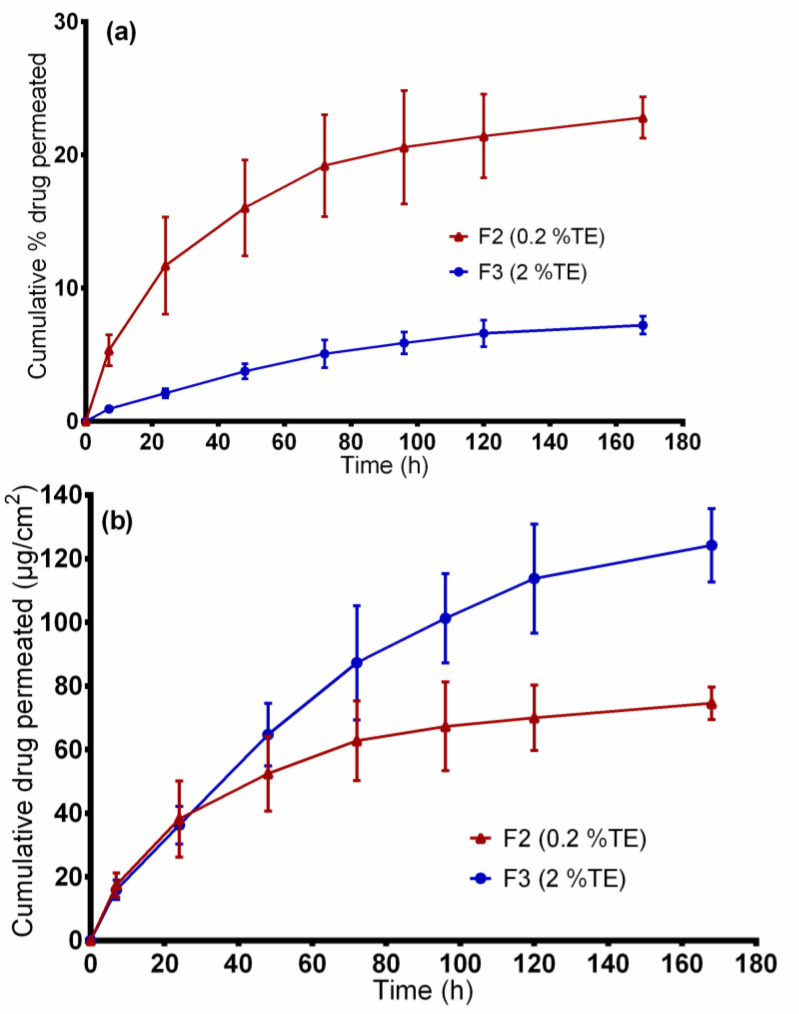
Ex vivo drug permeation studies. (**a**) percentage of permeated betulin and (**b**) amount of betulin permeated (µg/cm^2^) from TE-loaded wound dressings.

The developed wound dressings are intended to be applied on wounded skin. Figure 6 shows the cumulative amount of betulin permeated from the two wound dressings through stimulated “injured skin”. Again, the data is represented in both percent and µg/cm^2^. The cumulative amount of drug permeated from the F3 formulation was 124.24 ± 11.5 µg/cm^2^, and 74.58 ± 4.7 µg/cm^2^ for the F2 dressing at 168 h. The large differences in permeation profiles are due to the high amount of TE incorporated in the F3 formulation. However, the drug permeated much faster from the F2 dressing (permeation coefficient: 6.2 × 10^−3^ mg/cm^2^ × h), reaching about 22.81%, than the F3 formulation (permeation coefficient: 3.6 × 10^−3^ mg/cm^2^ × h) where only 7.21% was achieved at 168 h. Beyond that, as shown in Figure 6, the ex vivo drug permeation profiles were similar to in vitro drug-release profiles. On comparing the R^2^ values of the two formulations, again, betulin permeation profiles closely fitted to Korsmeyer–Peppas model (R^2^: 0.9914 for F2, 0.9934 for F3) and Higuchi square root model (R^2^: 0.9844 for F2, 0.9968 for F3) than other models. From the Korsmeyer–Peppas model, *n* values were found to be between 0.42 and 0.75 for both dressings, showing that betulin permeation from PVA/TE fiber mats can be mainly attributed to diffusion of the drug from the polymer matrix. Mostly, the release of the drug from drug-loaded electrospun nanofibers is mainly governed by diffusion through the polymer matrix, including release by polymer matrix degradation and drug desorption from the surface of the nanofibers [52,53].

As expected, in vitro drug release and skin permeation studies are markedly different (*p* < 0.05) in that the drug release with the use of synthetic membrane is much faster in comparison to the dermatomed skin. For instance, only 19% (62.78 ± 12.5 µg/cm^2^) of betulin from F2 permeated through the skin, whereas about 44% (145 ± 15.5 µg/cm^2^) was released through the synthetic membrane after 3 days. These data suggest that the diffusion of betulin across the synthetic membrane demonstrates how the skin due to its complexity, presents as a barrier leading to a slower permeation even when injured skin was tested.

### 3.6. Ex Vivo Wound Healing Model

**Figure 7:**
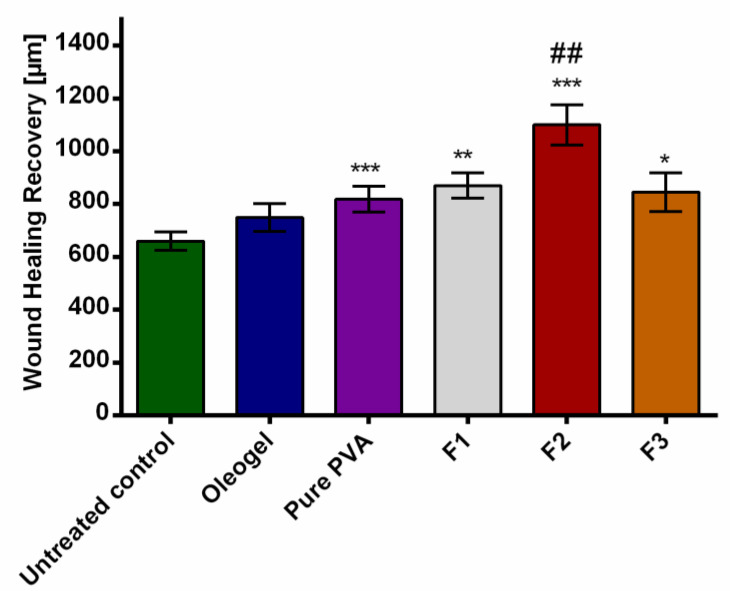
Wound healing recovery. * statistically significant, * *p* < 0.05; ** *p* < 0.01; *** *p* < 0.001 vs. untreated control and ## compared to TE-oleogel.

**Figure 8:**
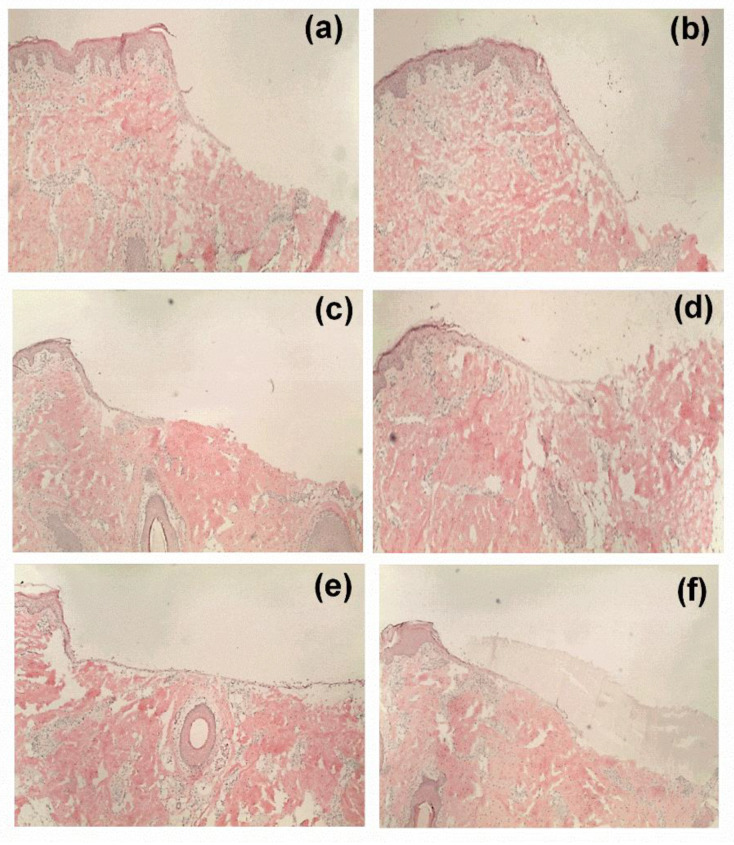
Microscopic observation: histological evaluations of wounds treated with TE-oleogel (**b**), pure PVA fiber mat (**c**), F1 (**d**), F2 (**e**), and F3 (**f**). (**a**) is the untreated control.

The healing performance of the wound dressings was microscopically evaluated in an ex vivo experiments. As shown in Figure 7, all the tested formulations favored wound closure in comparison to control, but the greatest wound area reduction was observed in the group treated with the F2 fiber mats. This is clearly reflected by the corresponding histological micrographs (Figure 8) where the F2 fiber mat (Figure 8e) showed a complete reepithelialization and proved to enhance wound healing more efficiently than all other tested samples. Statistical evaluation revealed that the differences with the rest of the groups were statistically significant (*** *p* < 0.001). Among the rest of the groups, the electrospun wound dressings performed much better than the TE-oleogel. Moreover, given that F2 fiber mats presented a significantly higher wound closure, it can be observed that low concentrations of TE are more favorable to wound healing than higher amounts of TE (F3 fiber mats, * *p* < 0.05). Perhaps higher concentrations of TE inside the fiber mats had some impairing effects on the cells, which slowed wound closure. In addition, the two placebos, F1 fiber mats (** *p* < 0.01) and pure PVA fiber mats (*** *p* < 0.001), also showed a significant improvement of reepithelialization in comparison to the control, but the formulation containing PL90H without TE was slightly better than pure PVA scaffolds. However, it would be interesting to assess the effects of PL90H on the wound healing process in a further study. Other researchers have shown that electrospun scaffolds in wound healing promote cell proliferation, cell support, and enhanced tissue regeneration [21,24].

## 4. Conclusions

In conclusion, for the first time, we have successfully developed a bioactive PVA-based wound dressing containing colloidal dispersions of birch bark extract through emulsion-electrospinning. The betulin was released from the electrospun fiber mats in a diffusion-controlled manner. Scanning electron microscopy showed that fiber characteristics and morphology can be influenced by just adjusting compositions in the colloidal dispersions. The electrospun fiber mats varied from smooth to rough surfaces with average fiber diameters between 300 and 1586 nm. Confocal Raman spectral imaging confirmed that TE and other substances were indeed part of the electrospun fiber structures. The ex vivo wound healing assay carried out in porcine ears showed an improvement in wound healing in terms of wound closure and reepithelialization when PVA-TE fiber mats were applied on the wound. Accordingly, these results demonstrated that the developed wound dressings ideally suited for the treatment of wounds. The proven sustained release enables less frequent changing of the wound dressing. Due to the extended TE release, application to chronic wounds seems also to be conceivable. Such a TE-PVA hybrid system comes with tremendous effects not only for covering wounds, but also essential in accelerating the wound healing process due to the positive effects of TE. In addition, the wound healing study showed that wound healing is a complex process and that the performance of a wound dressing can be not estimated neither from the physicochemical characterization nor from ex vivo permeation studies. Selection must be done on a case-by-case approach. However, it can be concluded that such a composite scaffold fabricated through emulsion electrospinning would be an appropriate candidate for wound healing applications.

## Figures and Tables

**Table 1:** Physicochemical characteristics of the dry birch bark extract used [9].

TE Composition	Specific Surface Area	Particle Size D50%
Betulin 81.60%, lupeol 2.08%, betulinic acid 3.84%, erythrodiol 1.05%, oleanolic acid 0.97%, Betulinic acid methyl ester 0.52%, unidentified substances 9.94%	42 ± 0.4 m^2^/g	5.8 µm

**Table 2:** Composition (wt.%) of the prepared aqueous dispersions with their particle sizes.

Dispersion	Composition	Particle Size (nm)
D1	2.5% PL90H, 1% SO and 0.5% TE	400 ± 49
D2	8% PL90H, 10% SO and 5% TE	840 ± 17
D3	8% PL90H, 10% SO and 0% TE	477 ± 58

**Table 3:** Final composition (wt.%) of formulation blends for electrospinning.

Component	Formula 1 (F1) {%}	Formula 2 (F2) {%}	Formula 3 (F3) {%}
PVA	7.2	7.2	7.2
PL90H	3.2	1	3.2
Sunflower oil	4	0.4	4
TE	-	0.2	2
Purified water	ad 100	ad 100	ad 100

**Table 4:** Fiber diameters of the obtained electrospun fibers.

Formulation	Average Diameter (nm)	Minimum Diameter (nm)	Maximum Diameter (nm)
Pure PVA	1105 ± 236	768	1586
F1	481 ± 49	399	577
F2	392 ± 42	341	499
F3	626 ± 94	390	853

**Table 5:** Thermal properties of nonloaded and TE-loaded electrospun PVA-based fiber mats.

Sample	T_m_ (°C)
Birch bark extract	248 ± 2.92
Phospholipon 90H	231 ± 1.11
Pure PVA mat	177 ± 0.42
F1	177 ± 0.27
F2	178 ± 1.53
F3	177 ± 0.14

**Table 6:** In vitro release kinetics data of the two TE-loaded electrospun fiber mats fitted to mathematical models.

Sample	Zero-Order	First-Order	Higuchi	Korsmeyer–Peppas Model	
	R^2^	R^2^	R^2^	R^2^	*n*
F2	0.9105	0.9405	0.9847	0.9943	0.42
F3	0.9834	0.9877	0.9975	1	0.62
