# Erratum: Mwiiri, F.K.; et al. Electrospun Bioactive Wound Dressing Containing Colloidal Dispersions of Birch Bark Dry Extract. *Pharmaceutics* 2020, *12*, 770

**DOI:** 10.3390/pharmaceutics12100991

**Published:** 2020-10-19

**Authors:** Francis Kamau Mwiiri, Johanna M. Brandner, Rolf Daniels

**Affiliations:** 1 Department of Pharmaceutical Technology, Eberhard Karls University, Auf der Morgenstelle 8, 72076 Tuebingen, Germany; f.kamau@gmx.de; 2 Department of Dermatology and Venerology, University Hospital Hamburg-Eppendorf, Martinistraße 52, 20246 Hamburg, Germany; brandner@uke.de

The authors wish to make the following correction to this paper [1]: In the experimental part, Section 2.4. Electrospinning of Nanofibers, the units of flow rate were 0.5 mL/h, but it was mistakenly written as 0.5 mL/min. The corresponding sentence has now been corrected in this erratum. 

“Solutions were pumped through the syringe at a flow rate of 0.5 mL/h and the rotating speed of the collector was fixed at 1000 rpm.”

The authors would like to apologize for any inconvenience caused to the readers by this change.
